# Exosomes derived from TNF-α preconditioned bone marrow mesenchymal stem cells alleviate cisplatin-induced ototoxicity in mice

**DOI:** 10.7150/ijms.104121

**Published:** 2025-02-18

**Authors:** Wei Li, Tao Yang, Zhiwen Zhang, Anquan Peng, Qin Wang

**Affiliations:** Department of Otolaryngology and Head & Neck Surgery, the Second Xiangya Hospital, Central South University, Changsha 410011, Hunan, China.

**Keywords:** Exosomes, TNF-α, bone marrow mesenchymal stem cells, cisplatin, ototoxicity

## Abstract

The polarization of microglia promotes the development of cisplatin-induced ototoxicity, and exosomes (Exo) derived from TNF-α preconditioned mesenchymal stem cells (MSCs) may induce the polarization of macrophage. Mice were intraperitoneally injected with cisplatin to establish the ototoxicity model. Bone marrow MSCs (BMSCs) were preconditioned with TNF-α for 48 h, and the relevant TNF-Exo or Exo was enriched, which were further trans-tympanically administered in the left ear of ototoxic mice. Auditory sensitivity was revealed with auditory brainstem response (ABR) at 8, 16, 24, and 32 kHz. The number of hair cells was detected with Myosin 7a staining. Damaged auditory sensitivity and up-regulated hair cell loss were revealed in cisplatin-exposed mice, which could be reversed by Exo or TNF-Exo treatment. Mechanically, up-regulated Iba1, Cd86, iNOS, Cd206, and Arg1 were detected in cisplatin-exposed cochlea. TNF-Exo or Exo administration further decreased Iba1, Cd86, and iNOS expression, and increased cd206 and Arg1 expression. TNF-Exo or Exo administration inhibited the productin of pro-inflammatory cytokines (IL-1β and IL-6), while enhanced the anti-inflammatory cytokine IL-10 production in the cisplatin-exposed cochlea. Importantly, TNF-Exo administration showed more profound benefits compared with Exo. TNF-α preconditioning might be a new therapeutic option to enhance the capability of BMSCs-derived exosomes against cisplatin-induced ototoxicity.

## Introduction

As the first inorganic platinum-based chemotherapeutics, cisplatin is generally utilized to treat various solid cancers 1, 2. Multiple mechanisms related to cancer cell apoptosis have been discovered. Among these, cisplatin can bind with DNA to form intrastrand cross-link conjugate to activate several signal transduction pathways, which induce apoptosis 3-5. Meanwhile, accompanying ototoxicity can happen and range from 20 to 70%, which might lead to temporary or permanent psychological impact 6-8. Mechanically, ototoxic drug treatment can alter the number, morphology, and differentiation of macrophages 9, 10, and macrophage-related inflammation contributes to progressive hair cell death and consequent ototoxicity 11. All of these indicate that targeting cisplatin-induced inflammation-related ototoxicity is emergent in clinical practice.

In multiple preclinical studies, the potential to utilize exosomes derived from bone marrow mesenchymal stem cells (BMSCs) to alleviate ototoxicity is investigated, when considering their high delivery efficiency, biocompatibility, and multifunctional characteristics 12, 13. More evidence indicates that exosomes derived from preconditioning BMSCs show more treatment benefits than traditional exosome administration 14, 15. Tumor necrosis factor-alpha (TNF-α) is a pro-inflammatory cytokine that mimics the inflammatory milieu often present in pathological conditions 16. Pre-treating MSCs with TNF-α equips them to better respond to inflammatory stimuli *in vivo*, tailoring their therapeutic effects to the disease environment 17, 18. Under physiological conditions, BMSCs can secret a low level of TNF-α, which is required for self-renewal and differentiation of BMSCs *via* autocrine/paracrine signaling 19, 20. TNF-α exposure increases the secretion of key immunosuppressive factors by MSCs 21. In inflammatory bowel disease, TNF-α can enhance the therapeutic effects of exosomes derived from MSCs through the induction of macrophage polarization 22. All of these indicate the potential to utilize exosomes derived from TNF-α preconditioning BMSCs to alleviate ototoxicity.

In this study, we aim to study the effects of exosomes derived from TNF-α preconditioning BMSCs (TNF-Exo) on cisplatin-induced ototoxicity, focusing on microglia polarization and inflammatory cytokines microenviroment in the cochlea.

## Methods & materials

### TNF-α preconditioned BMSCs

BMSCs isolation was performed as indicated in our previous research 13, 15. C57BL mice (6-8 weeks) were ordered from Peking Vital River Laboratory Animal Ltd. (Beijing, China). Bone marrow cells were flushed out from the femurs, which were further centrifuged (800 rpm, 5 min) and enriched with CD11b microbeads (StemCell Technologies) to obtain BMSCs. BMSCs were cultured in Corning MEM medium with 10% exosome-depleted fetal bovine serum (Gibco) and seeded into six-well dishes (5×10^3^ cells/cm^2^). Flow cytometry was utilized to testify BMSCs with the detection of CD105, CD29, CD45, and CD34 (data not shown). TNF-α (Biolegend, 20 ng/ml) was utilized to incubate the 3rd passage of BMSCs for 48 hours.

### Exosome isolation and characterization

The media derived from TNF-α preconditioned BMSCs or normal cultured BMSCs were centrifuged (350× g, 15 min, 4°C; 16,000 × g, 30 min, 4°C), filtered with Millipak 0.22 µm filter, and ultra-centrifuged (120,000 × g, 70 min, 4°C) to get exosome suspension, which was resuspended with physiological saline.

TNF-Exo or Exo size distribution was assayed with nanoparticle tracking in NanoSight NS300 equipment. The surface markers of exosome (CD9, CD63, Tsg101, and Alix) were assayed with Western blots, and transmission electron microscopy (TEM) was utilized to reveal the membrane structure with a JEM-1400Flash Electron Microscope (Jeol, Tokyo, Japan).

### Cisplatin-exposed mice and exosomes treatment

A single intraperitoneal injection of 1 mg/mL cisplatin (30 mg/kg) was performed on C57BL/6 mice (6-8 weeks, n=10). After half an hour, 1.2 µg/µL TNF-Exo or Exo (1 μL) was trans-tympanically introduced in the left ear. Sham surgery was performed on the control group with the intraperitoneal administration of physiological saline. The whole protocol was approved by the Ethics Committee of Xiangya Hospital.

### Auditory brainstem response (ABR)

Cisplatin-exposed mice were euthanized one week after cisplatin exposure, and the TDT System III apparatus (Tucker Davies Technologies) was utilized to detect ABR. In brief, acoustic stimuli (frequencies: 8, 16, 24, and 32 kHz; intensities: 10-100 dB; duration: 100 ms) was administrated into the ear canal. Detected wave V was identified as a hearing threshold. Dissociated cochlear tissues were used for relative content and immunofluorescence detection after ABR measurement.

### Quantitative real-time PCR

PureLink RNA Mini Kit was adopted to extract total RNA from the pooled cochlea tissues. Applied Biosystems High-Capacity cDNA Reverse Transcription Kit was utilized to reverse-transcribe total RNA into cDNA. Further amplification was detected with SYBR Green (Roche) with the following protocol: 95°C, 10 min; 95°C, 15 s, 35 cycles; and 60°C, 1 min. Primer sequences were listed: Cd86, 5'-TCAATGGGACTGCATATCTGCC-3', reverse primer 5'-GCCAAAATACTACCAGCTCACT-3'; Cd206, forward primer 5'-GGGACTCTGGATTGGACTCA-3', reverse primer 5'-GCTCTTTCCAGGCTCTGATG-3'; Iba1, forward primer 5'-CTTGAAGCGAATGCTGGAGAA-3', reverse primer 5'-GGCAGCTCGGAGATAGCTTT-3'; and Gapdh, forward primer 5'-TTTGCACTGGTACGTGTTGAT-3', reverse primer 5'-AATGGATTTGGACGCATTGGT-3′. The relative expression was quantified with the 2^-ΔΔCt^ method after normalizing to Gapdh.

### Western blotting

The cochlear tissues, Exo, and TNF-Exo lysates were separated with 10% SDS-PAGE and transferred to polyvinylidene fluoride membranes. Primary antibodies against CD63, CD9, Alix, Tsg101, Bax, Bcl2, Iba1, Cd86, iNos, Cd206, Arg1, Cleaved caspase3, and Gapdh (Santa Cruz, 1:1000 dilution, overnight) and peroxidase-conjugated secondary antibody (Santa Cruz, 1:3000 dilution, two hours) were sequencing incubated. The signal was developed with a Pierce Enhanced Chemiluminescence. GAPDH was utilized as an internal control, and the relative expression was analyzed with NIH-Image J1.51p 22.

### Myosin staining

Paraformaldehyde (4%) was used to fix cochlea tissues and Triton X-100 (1%) was adopted to permeabilize fix cochlea tissues (2-hour, at room temperature). The permeabilized cochlea tissues were incubated with primary anti-myosin 7a antibody conjugated with FITC (Santa Cruz). The percentage of hair cells was indicated with the number of Myo7a-positive cells/100 μm. Representing images were observed with a Nikon 80*i* microscope.

### Enzyme-linked immunosorbent assay (ELISA)

The relative content of IL-1β, IL-10, and IL-6 was detected with relevant ELISA kits (eBioscience) according to the manufacturer's instructions. All standard solutions and samples were assayed with a SpectraMax M5 microplate reader (Molecular Devices) at a wavelength of 450 nm.

### Statistical analysis

Brown-Forsythe ANOVA test followed by Dunnett's T3 multiple comparisons test were utilized to reveal the differences between groups. The significance level was set at a *p*-value < 0.05. All statistical analyses were performed with GraphPad Prism.

## Results

### Characterization of TNF-α preconditioned BMSC-derived exosomes

**Figure 1:**
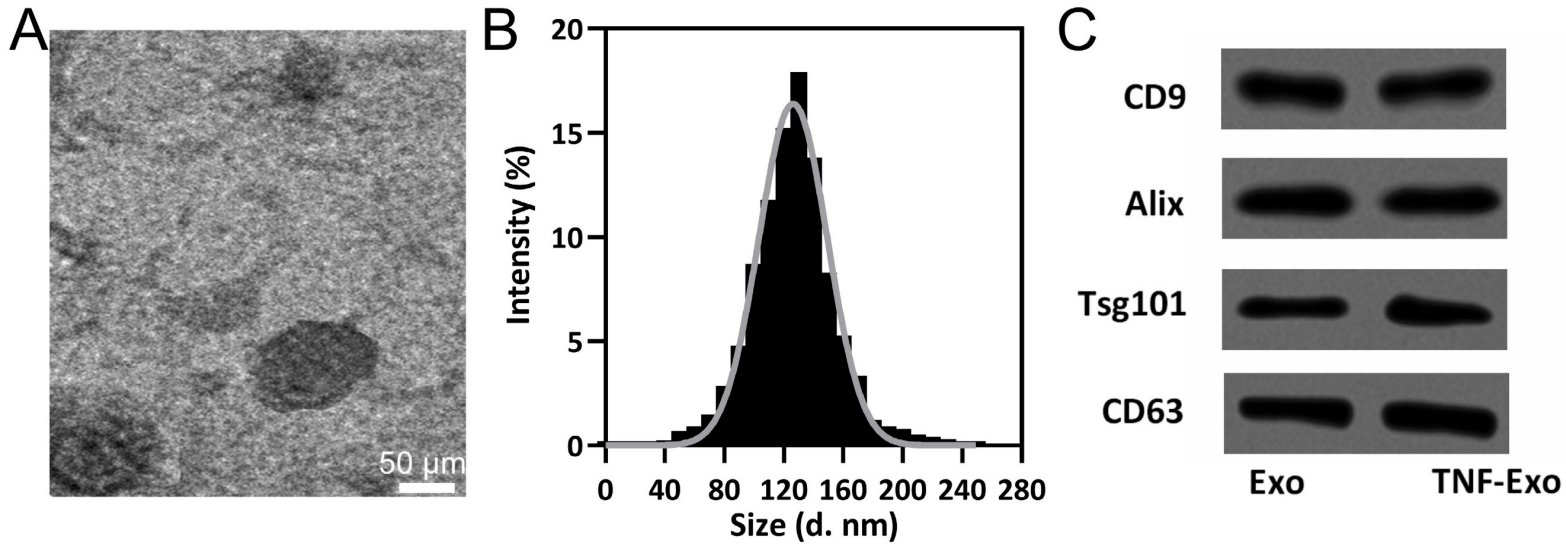
BMSCs derived exosomes characterization. A, Representative TEM image of exosomes derived from TNF-α preconditioned BMSCs (TNF-Exo). B, The size distribution of TNF-Exo revealed by nanoparticle tracking analysis. C, CD9, Alix, Tsg101, and CD63 detection by Western blotting from exosomes derived from BMSCs (Exo) and TNF-Exo.

TEM (Figure 1A), size distribution (Figure 1B), and exosomal markers detection (Figure 1C) were performed on TNF-Exo and Exo to identify the influence of TNF-α precondition on the characteristics of exosomes. Our results demonstrated that TNF-Exo and Exo demonstrated similar structures (single membrane), size distribution (average:110-120 nm), and relative expression of CD9, Alix, Tsg101, and CD63. All in all, TNF-α precondition did not influence the characterization of exosomes derived from BMSCs.

### TNF-Exo ameliorates auditory sensitivity in ototoxic mice

**Figure 2:**
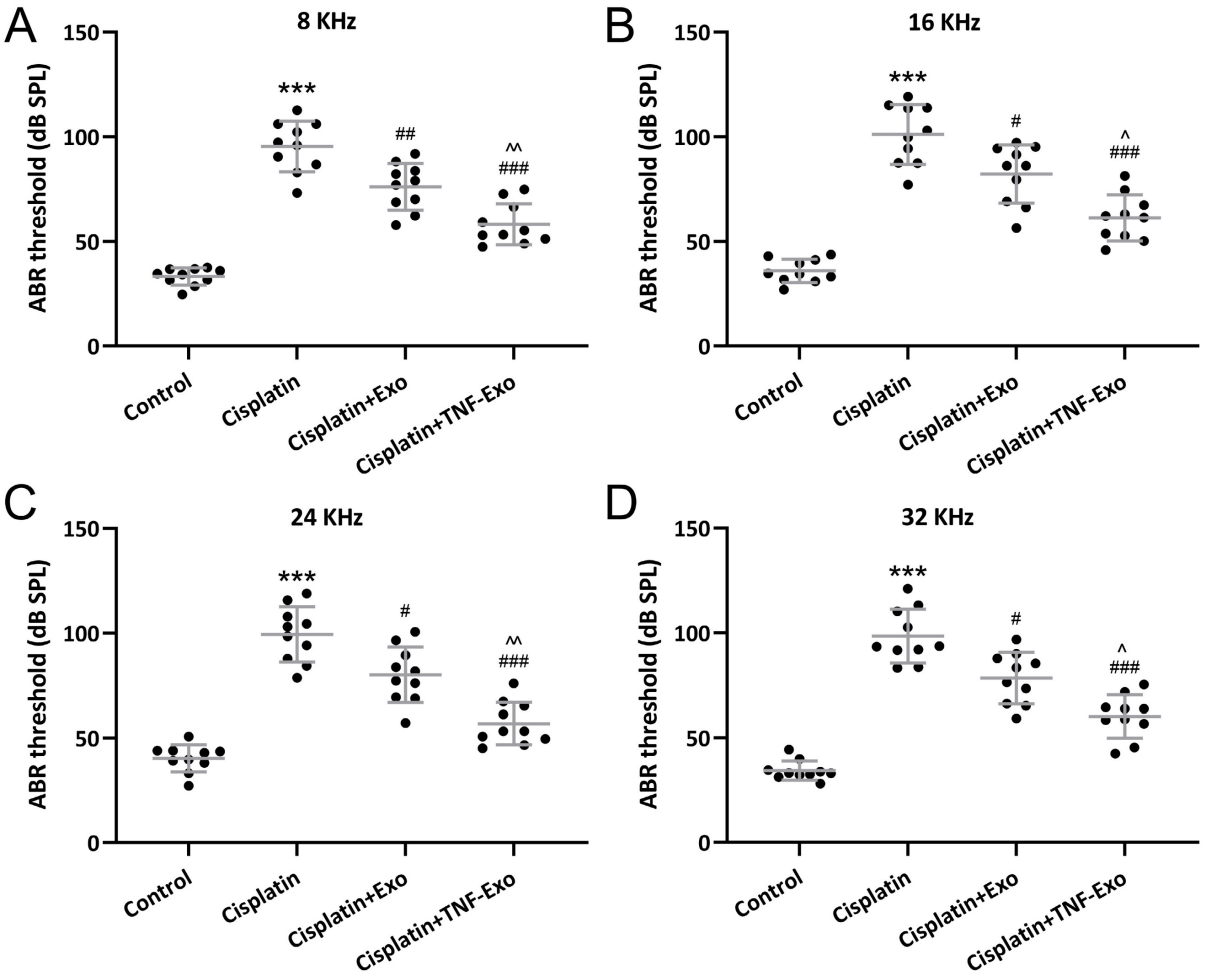
TNF-Exo ameliorated auditory sensitivity in ototoxic mice. A-D, ABR detection (at 8, 16, 24, and 32 kHz) after Exo or TNF-Exo treatment in cisplatin exposed mice (n = 10). ***p < 0.001 compared to Control group, #*p* < 0.05, ##*p* < 0.01, ###*p* < 0.001 compared to Cisplatin group, ^*p* < 0.05, ^^*p* < 0.01 compared to Cisplatin+Exo group. Brown-Forsythe ANOVA test followed by Dunnett's T3 multiple comparisons test.

Auditory sensitivity was revealed with ABR measurement. Cisplatin exposure greatly elevated the hearing thresholds in the cisplatin-exposed mice at 8 kHz (Control: 33.28±4.14 dB; Cisplatin: 95.42±12.14 dB) (Figure 2A), 16 kHz (Control: 35.98±5.61 dB; Cisplatin: 101.10±14.29 dB) (Figure 2B), 24 kHz (Control: 40.31±6.51 dB; Cisplatin: 99.38±13.23 dB) (Figure 2C), and 32 kHz (Control: 34.34±4.60 dB; Cisplatin: 98.52±12.79 dB) (Figure 2D), whereas Exo or TNF-Exo administration could down-regulate the thresholds significantly. When compared with Exo (6 kHz: 76.13±11.13 dB; 16 kHz: 82.19±13.98 dB; 24 kHz: 80.14±13.25 dB; 32 kHz: 78.42±12.27 dB), TNF-Exo dramatically decreased the elevated hearing thresholds at the detected four frequencies (6 kHz: 58.25±9.81 dB; 16 kHz: 61.28±11.08 dB; 24 kHz: 56.87±10.12 dB; 32 kHz: 60.14±10.34 dB). All of these indicated that TNF-Exo restored auditory sensitivity in ototoxic mice and demonstrated more therapy benefits than Exo.

### TNF-Exo ameliorates hair cell loss in ototoxic mice

**Figure 3:**
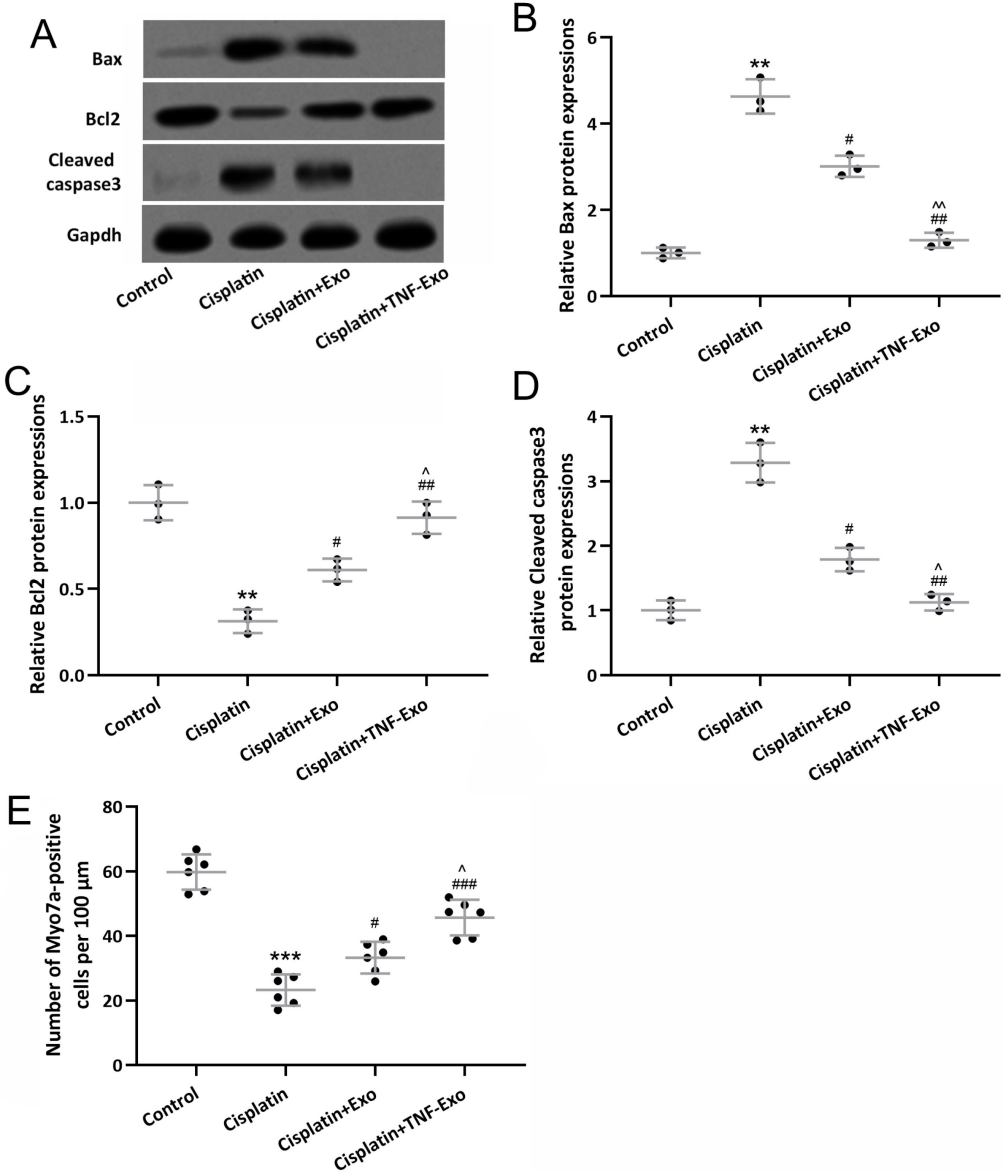
TNF-Exo inhibited cisplatin-induced hair cell loss. A, Western blotting was adopted to detect the cochlear Bax, Bcl2, and cleaved caspase3 expression from different groups. The expression was normalized to control (B-D, n=3). E, Myosin 7a-positive hair cells quantification (n=6) in the cochlea. **p < 0.01, ***p < 0.001 compared to Control group, #*p* < 0.05, ##*p* < 0.01, ###*p* < 0.001 compared to Cisplatin, ^*p* < 0.05, ^^*p* < 0.01 compared to Cisplatin+Exo. Brown-Forsythe ANOVA test followed by Dunnett's T3 multiple comparisons test.

In order to reveal the treatment benefit of TNF-Exo in the cochlea, the relevant apoptosis protein was detected with Western Blot (Figure 3A). Cisplatin exposure up-regulated Bax and cleaved caspase 3 expression (Figure 3B and 3D), and down-regulated Bcl2 expression (Figure 3C), which could be reversed by Exo or TNF-Exo therapy. It was noteworthy that the TNF-Exo group showed a more significant effect on the alteration of apoptosis protein expression when compared with the Exo group.

A highly organized cochlea structure indicates functional auditory function. Our results demonstrated that a significant loss of hair cells was detected after cisplatin exposure (Control: 59.73±5.45 cells per 100 μm; Cisplatin: 23.24±4.84 cells per 100 μm), and TNF-Exo and Exo administration alleviated the loss of hair cells (Figure 3E). Compared with Exo (33.23±4.91 cells per 100 μm), TNF-Exo treatment significantly alleviated hair cell loss (45.64±5.53 cells per 100 μm) (*p*<0.05). All of these testified that TNF-Exo prevented hair cell loss with diminished apoptosis in ototoxic mice.

### TNF-Exo modulates microglial activation and polarization in ototoxic mice

**Figure 4:**
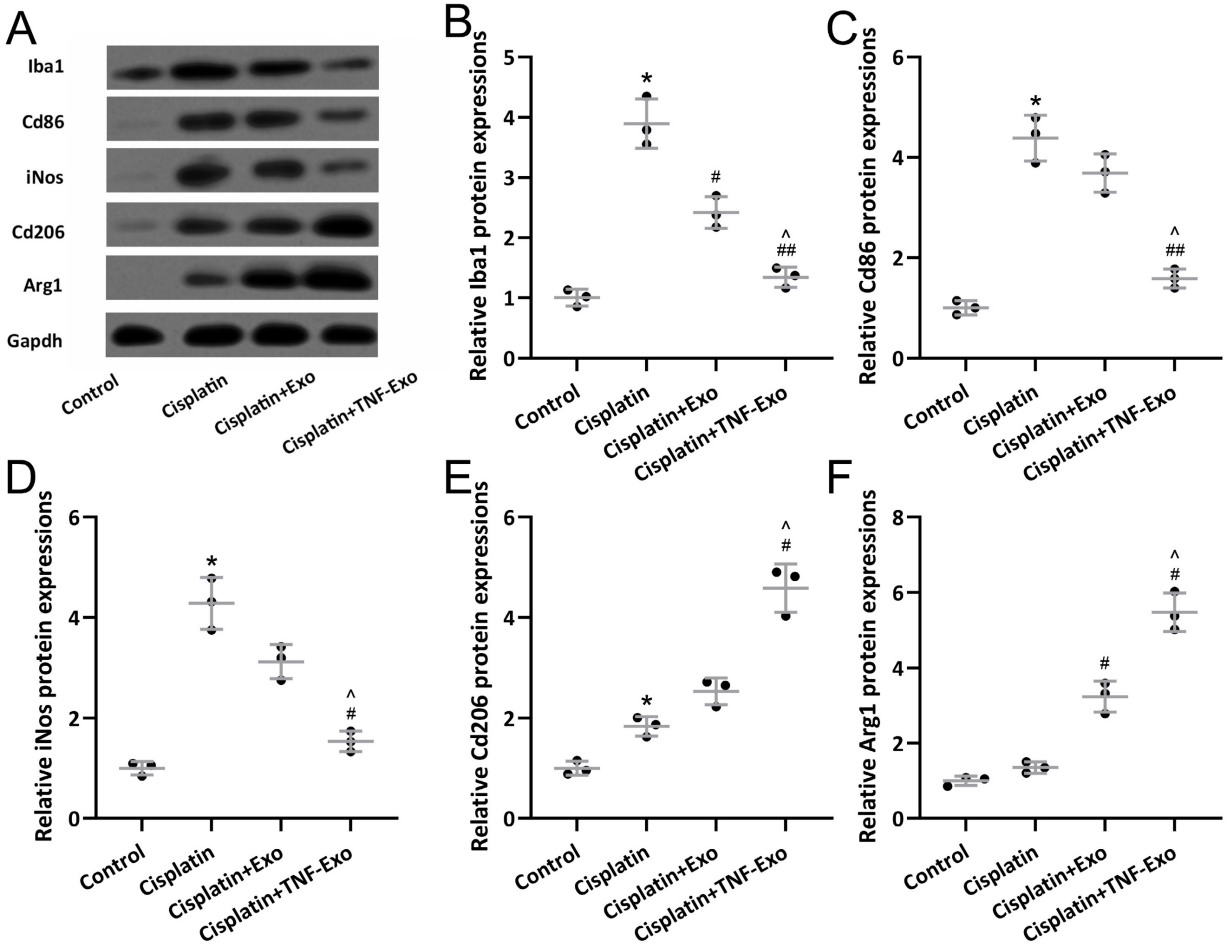
TNF-Exo modulated microglial activation and polarization in the cochlear after cisplatin treatment. Iba1, Cd86, iNos, CD206, and Arg1 in the cochlea were measured with Western blotting. The expressions were normalized to Control (B-F, n = 3). *p < 0.05 compared to Control group, #*p* < 0.05, ##*p* < 0.01 compared to Cisplatin group, ^*p* < 0.05 compared to Cisplatin+Exo group. Brown-Forsythe ANOVA test followed by Dunnett's T3 multiple comparisons test.

Western blotting was adopted to detect microglia M1 markers (Iba1, Cd86, iNos) and M2 markers (CD206, Arg1) expression in the cisplatin-exposed cochlea (Figure 4A). Up-regulated Iba1 (Figure 4B), Cd86 (Figure 4C), iNOS (Figure 4D), Cd206 (Figure 4E), and Arg1 (Figure 4F) were detected in cisplatin-exposed cochlea. TNF-Exo or Exo administration decreased the expression of Iba1, Cd86, and iNOS, and increased the expression of cd206 and Arg1. Significantly, TNF-Exo administration showed more treatment benefits compared with Exo.

### TNF-Exo modulates microglial M1/M2 polarization in ototoxic mice

**Figure 5:**
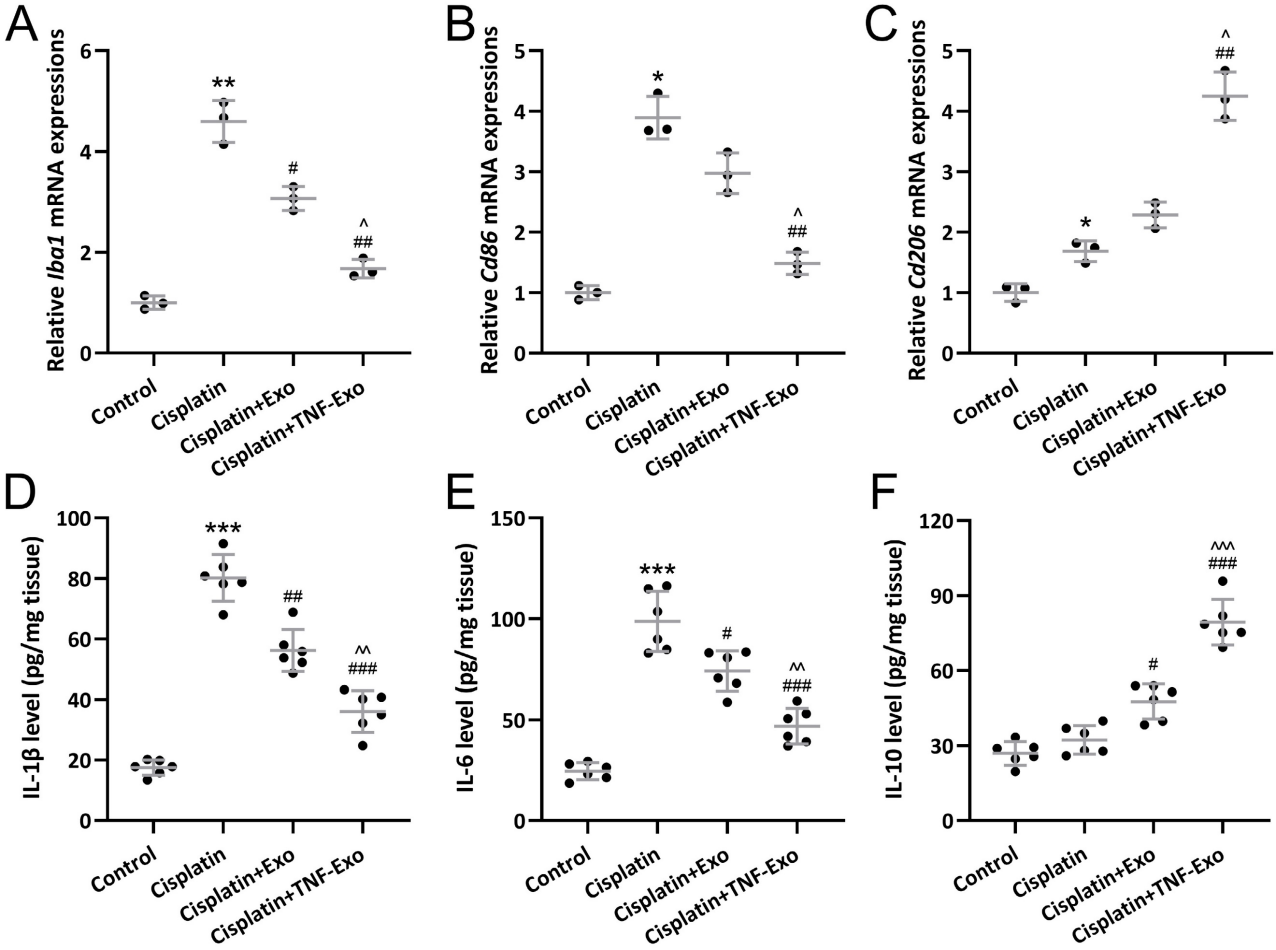
Exosomes derived from TNF-α treated BMSCs modulated microglial M1/M2 polarization mediated inflammatory responses in the cochlear after cisplatin treatment. qRT-PCR was adopted to assay Iba1 (A), Cd86 (B), Cd206 (C) expression in cochlea tissues (n = 3). ELISA was utilized to detect the concentrations of IL-1β (D), IL-6 (E), and IL-10 (F) in cochlea homogenates (n = 6). *p < 0.05, **p < 0.01, ***p < 0.001 compared to control group, #*p* < 0.05, ##*p* < 0.01, ###*p* < 0.001 compared to Cisplatin group, ^*p* < 0.05, ^^*p* < 0.01, ^^^*p* < 0.001 compared to Cisplatin+Exo group. Brown-Forsythe ANOVA test followed by Dunnett's T3 multiple comparisons test.

On the other hand, qRT-PCR was utilized to detect Iba1, Cd86, and Cd206 expression in cochlea tissues. As expected, up-regulated Iba1 (Figure 5A), Cd86 (Figure 5B), and Cd206 (Figure 5C) were observed in cisplatin-exposed cochlea. TNF-Exo or Exo administration could decrease Iba1 and Cd86 expression, and increase cd206 expression.

On the other hand, ELISA was adopted to detect cytokines content in cochlea homogenates. Up-regulated IL-1β (Control: 17.47±2.54 pg/mg tissue; Cisplatin: 80.18±7.71 pg/mg tissue) (Figure 5D) and IL-6 (Control: 24.55±4.20 pg/mg tissue; Cisplatin: 98.74±14.92 pg/mg tissue) (Figure 5E) were detected in the cisplatin-exposed cochlea, which could be significantly reduced by TNF-Exo (IL-1β: 36.03±6.85 pg/mg tissue; IL-6: 46.85±8.81 pg/mg tissue) or Exo (IL-1β: 56.20±6.94 pg/mg tissue; IL-6: 74.14±9.98 pg/mg tissue) administration. On the other hand, TNF-Exo or Exo administration up-regulated the secretion of IL-10 in the cisplatin-exposed cochlea (Control: 26.95±4.72 pg/mg tissue; Cispaltin: 32.28±5.73 pg/mg tissue: Exo: 47.64±6.97 pg/mg tissue; TNF-Exo: 79.30±9.10 pg/mg tissue) (Figure 5F). These results confirmed that TNF-Exo could modulate microglial M1/M2 polarization in ototoxic mice.

## Discussion

TNF-Exo alleviates cisplatin-induced ototoxicity with improved hair cell loss, up-regulated auditory sensitivity, inhibited inflammation cytokines release, and up-regulated M2 polarization when compared with Exo. TNF-α precondition induced BMSCs-derived exosomes are proposed as a novel therapeutic agent to promote regeneration and immunomodulation of cisplatin-induced ototoxicity. Our data also indicates that TNF-α pre-conditioning methods can be considered as a strategy for improving the therapeutic efficacy of BMSCs.

Exosomes derived from BMSCs preserve the therapeutic potential of the parent BMSCs, and the administration of exosomes could avoid the safety concern associated with live cell therapy 23-25. It is testified that BMSCs exosomal contents and functions are greatly influenced by the microenvironment. Ischemic tissue simulated conditions exposed BMSCs demonstrate up-regulated proangiogenic factors, which can be utilized to treat ischemic diseases 26. On the other hand, exosomes derived from hypoxia-preconditioned MSCs exhibit greater therapeutic effects on femoral fracture healing 27. In chemotherapy-induced premature ovarian failure, heat shock precondition can enhance the repair effect of mesenchymal stem cells by inhibiting the apoptosis of ovarian granulosa cells 28. In this study, we further demonstrate that TNF-α preconditioned BMSCs could alter the polarization of microglia in the cochlea with diminished proinflammation cytokines expression.

As highly specialized tissue macrophages in the cochlea, microglia contribute to the development and cleaning of the auditory system 29. In the developing auditory system, microglia are activated and subsequently polarized into pro-inflammatory (M1) or anti-inflammatory (M2) phenotypes, and the phenotypic transition might lead to tissue remodeling or homeostasis after early hearing loss 24, 30. Mounting evidence has testified that modulation microglia polarization is a potential target approach to treat ototoxicity. In this study, microglia functional plasticity in the cochlea is modulated by TNF-α preconditioned BMSCs-derived exosomes. Whether TNF-Exo could be utilized to affect other central nervous system diseases is an interesting question needed to be answered.

Some limitations should also be indicated here. Cisplatin-induced ototoxicity usually happen in the early stages of exposure (from hours to days), leading to progressive, and cumulative hearing loss. In this study, only the short-time (7-day) treatment benefit of TNF-Exo is observed after cisplatin exposure, and the chronic treatment benefit is not deciphered in this study. The standard TNF-Exo preparation process should be developed to improve the production of TNF-Exo. The content of TNF-Exo and the following therapy mechanism should be deciphered in the future.

## Conclusion

Exosome therapy holds significant clinical value in combating cisplatin-induced ototoxicity. Exosomes derived from MSCs or other sources can carry bioactive molecules such as microRNAs, proteins, and lipids that modulate oxidative stress, inflammation, and apoptosis—key contributors to cisplatin-induced damage in the cochlea. By delivering these therapeutic agents directly to damaged tissues, exosomes can promote cellular repair, protect sensory hair cells, and preserve hearing function. Their nanoscale size, biocompatibility, and ability to cross biological barriers make exosomes a promising non-invasive treatment modality with reduced risks compared to systemic pharmacological approaches. Our results suggest that the efficacy could be further enhanced by pretreated the MSCs with TNF-α. The current findings lay the foundation for potential clinical applications of TNF-Exo in mitigating ototoxic effects.

## Author declarations

### Funding

This work was supported by the National Science Foundation of China (No. 82002895, No. 81600811); Changsha Natural Science Foundation (No. kq2202411); the Natural Science Foundation of Hunan Province, China (No. 2022JJ30844), and Clinical Medical Research Center for Otology in Hunan Province (2023SK4030).

### Ethics approval and consent to participate

The whole protocol was approved by the Ethics Committee of the Second Xiangya Hospital, Central South University.

### Availability of data and material

The raw data supporting the conclusions of this article will be made available by the authors, without undue reservation.
